# The Role of Gene Duplication in the Divergence of Enzyme Function: A Comparative Approach

**DOI:** 10.3389/fgene.2021.641817

**Published:** 2021-07-14

**Authors:** Alejandro Álvarez-Lugo, Arturo Becerra

**Affiliations:** ^1^Posgrado en Ciencias Biológicas, Universidad Nacional Autónoma de México, Mexico City, Mexico; ^2^Facultad de Ciencias, Universidad Nacional Autónoma de México, Mexico City, Mexico

**Keywords:** gene duplication, enzymatic classes, paralogous enzymes, enzyme evolution, function class

## Abstract

Gene duplication is a crucial process involved in the appearance of new genes and functions. It is thought to have played a major role in the growth of enzyme families and the expansion of metabolism at the biosphere’s dawn and in recent times. Here, we analyzed paralogous enzyme content within each of the seven enzymatic classes for a representative sample of prokaryotes by a comparative approach. We found a high ratio of paralogs for three enzymatic classes: oxidoreductases, isomerases, and translocases, and within each of them, most of the paralogs belong to only a few subclasses. Our results suggest an intricate scenario for the evolution of prokaryotic enzymes, involving different fates for duplicated enzymes fixed in the genome, where around 20–40% of prokaryotic enzymes have paralogs. Intracellular organisms have a lesser ratio of duplicated enzymes, whereas free-living enzymes show the highest ratios. We also found that phylogenetically close phyla and some unrelated but with the same lifestyle share similar genomic and biochemical traits, which ultimately support the idea that gene duplication is associated with environmental adaptation.

## Introduction

Gene duplication is one of the most important mechanisms that lead to the appearance of new genes and new functions (Ohno, 1970) in both prokaryotes (Serres et al., 2009; Wang and Chen, 2018) and eukaryotes (Maere et al., 2005; Panchy et al., 2016). There are distinct categories of duplications: those that comprise one or few genes (small-scale duplication; SSD) and those that comprise many genes (large-scale duplication; LSD) or even the entire genome (whole-genome duplication; WGD). SSDs have been widely documented in both prokaryotes and eukaryotes (Conant and Wagner, 2002). On the other hand, LSDs, specifically WGDs, for a long time had been considered to be an exclusively eukaryotic trait, but recent evidence strongly suggests that it is much prevalent in prokaryotes than we have previously thought (Pecoraro et al., 2011; van de Peer et al., 2017) and that it might be a way to cope with extreme environmental conditions (Soppa, 2017).

Theoretically, almost every gene has a similar probability of being duplicated, but not all are equally retained (McGrath et al., 2014). Most duplicated genes are eventually silenced in the short term (Lynch and Conery, 2000), and those that remain can either retain the original function (Zhang, 2003) or acquire a new one, either by subfunctionalization (a subdivision of an ancestral, often generalist function) or neofunctionalization (acquisition of a novel function) (Walsh, 2003). Besides providing the raw material for the emergence of new gene functions, gene duplication also seems to play an essential role in the adaptation of organisms to different environments (Gevers et al., 2004; Bratlie et al., 2010; Kondrashov, 2012) and in more complex processes like species diversification and increases in biological complexity (van de Peer et al., 2009).

Gene duplication has been a widespread mechanism in the evolution of metabolism. The Patchwork model (Yčas, 1974; Jensen, 1976), which is perhaps the most accepted model for metabolic evolution, suggests that gene duplication may have played a crucial role at the dawn of metabolism. At this stage, ancient enzymes probably lacked substrate or reaction specificity, allowing them to catalyze different reactions involving more than one substrate. Over time, one or more of these ancestral activities could have become so important that the ancestral enzyme could not have carried them out in the most efficient way. Thus, a duplication event involving such an enzyme could have led to a new copy with increased specificity. According to this model, throughout evolution, different metabolic pathways could have been assembled from the recruitment of newly evolved enzymes (Lazcano and Miller, 1999; Schmidt et al., 2003; Caetano-Anollés et al., 2009; Fani and Fondi, 2009; Becerra, 2021). Evidence of episodes of gene duplication leading to the enrichment of metabolic functions is found in both ancient and recent metabolic innovations. For example, it has been suggested that around three billion years ago, in a period known as the Archaean genetic expansion, gene duplication contributed to the appearance of new genes involved in respiratory and electron-transport pathways (David and Alm, 2011). It also seems to have fostered the expansion of many secondary metabolic pathways in plants (Weng et al., 2012; Moghe et al., 2017). Moreover, even for recently evolved pathways, like the mandelate pathway in several *Pseudomonas* species (Petsko et al., 1993), there is compelling evidence suggesting that some of the enzymes involved may have arisen by gene duplication.

It is now generally assumed that early life could have done well with a very limited set of enzymatic functions (Goldman et al., 2012), which could serve as a starting point for the evolution of new functions through scenarios involving gene duplication and other mechanisms like domain combinations, which could also lead to the appearance of functions other than catalytic activity (Bashton and Chothia, 2007). It has also been suggested that an interplay between the patchwork and the retrograde evolution model (Horowitz, 1945) is more likely than either of the two separately (Díaz-Mejía et al., 2007). Today, we can observe the outcome of these processes in the great functional diversity found within families and superfamilies of enzymes, at the levels of catalytic machinery, substrate specificity, and reaction chemistry (Bartlett et al., 2003; Furnham et al., 2016), though it is more common to see a greater substrate diversity within a single superfamily (Todd et al., 2001). Additionally, it is quite common to see drastic functional changes across the evolutionary history of enzymes. This is illustrated by the fact that changes in enzymes’ primary function (defined by the first digit of the Enzyme Commission number) have been observed between every enzymatic class, though some are more frequent than others (Furnham et al., 2012; Martínez Cuesta et al., 2015). But ultimately, what seems to be more important for the appearance of new functions is the inherent capacity of an enzyme to accept different substrates and/or perform different reactions (known as substrate and catalytic promiscuity, respectively) and its ability to evolve new functions in a changing environment (Tyzack et al., 2017).

The current enzyme classification system, which assigns a unique four-digit number for each enzyme, is exclusively based on the biochemical activities performed by each enzyme and groups them in terms of reaction similarity (McDonald et al., 2015), and not by evolutionary-related members. It was established during the early 60s by the International Commission on Enzymes from the International Union of Biochemistry and Molecular Biology (Tipton and Boyce, 2000). Until the first half of 2018, the classification remained without significant changes and consisted of six enzymatic (EC) classes, divided into different sub and sub-subclasses (McDonald and Tipton, 2014). However, in the second half of 2018, a new enzymatic class was added: the translocases (EC 7). A statement made in the ExplorEnz database (McDonald et al., 2009) highlighted the importance of a group of enzymes whose main function is the movement of ions or molecules from one side of biological membranes to the other. Many of these perform a different reaction as a means of achieving the movement of substances across membranes.

In this work, we try to analyze the role of gene duplication in the diversification of enzymatic functions across the enzymatic classes of the Enzyme Commission (EC) classification, including the recently proposed translocases (EC 7). We further explore the possible link between organisms’ lifestyle and specific patterns of retention of duplicated enzymes. Besides, due to recent proposals of a two-domain view of life, which suggests that eukaryotes do not constitute a separate domain but are part of the Archaea domain (Williams et al., 2013; Doolittle, 2020), we decided only to consider prokaryotic organisms, which as a group possess a much wider biochemical repertoire than that for eukaryotes.

## Materials and Methods

### Proteomes Analyzed

The complete set of prokaryotic proteomes was downloaded from the KEGG Database (Kanehisa and Goto, 2000). We selected a sample of non-redundant, representative proteomes based on criteria reported elsewhere (Martínez-Núñez et al., 2013, 2015). Altogether, we analyzed 655 bacterial and 90 archaeal proteomes (Supplementary Data Sheet 2). These belong to organisms whose genome has been completely sequenced, except for those from the phyla Bathyarchaeota and Lokiarchaeota, which come from metagenomic sequences.

### Identification of Within-Genome Paralogous Sequences

For this work, the criteria for defining paralogous proteins included an *E*-value cutoff of 10e-07 and query coverage ≥70%. We performed an *all against all* BlastP search (Altschul et al., 1997) for each of the 745 proteomes from the sample. Different Perl *ad hoc* scripts were used to filter the BlastP results and retain only those sequences that fulfilled the above criteria.

### Identification of Enzymes

Once we filtered the BlastP results, we extracted the IDs from the proteomes and paralogous data sets and crosschecked them with the FTP files downloaded from the KEGG Database. Additionally, the online tool db2db, from the bioDBnet resource (Mudunuri et al., 2009), was used to corroborate the enzyme codes (EC numbers) for all the paralogous-enzyme sequences. These are taken directly from the KEGG database. We considered all the sequences for which we obtained, at least, the first digit from the EC number, which refers to the general function of the enzyme (Tian and Skolnick, 2003; Concu and Cordeiro, 2019). Sequences for which we did not obtain an EC number were excluded from the subsequent analysis. EC codes from translocases (EC 7) had not been properly updated in the db2db tool. To solve this problem, we identified which enzymes had changed their EC code and manually updated them.

### Ratio of Paralogous Enzymes

We counted the number of enzymes and sorted them into one of the seven enzymatic classes for each of the proteomes and their respective paralogous data sets. The ratio of paralogous enzymes per class was defined as the ratio between the number of paralogous enzymes and the number of enzymes found within the proteome. In sum, we obtained seven different ratios per organism.

### Statistical Analysis

Non-parametric Kruskal–Wallis tests were used to evaluate the difference between paralogous enzymes for all enzymatic classes, followed by Dunn tests with the Bonferroni adjustment to identify those classes which differed significantly. Additionally, Spearman’s test was used to evaluate the relationship between the number of proteins and the number of enzymes, and a number of different regression analyses were also performed. In all cases, statistical significance was set at *p* ≤ 0.05. All statistical analyses were done with the R programming language (R Core Team, 2020) in the RStudio software (RStudio Team, 2020).

### Lifestyles Identification

After selecting our representative sample, we assigned the lifestyle to each of the organisms in our set. Such lifestyles are free-living, extremophile, pathogen, and intracellular. We relied on data from Martínez-Núñez et al. (2013) and the prokaryotic metadatabase BacDive (Reimer et al., 2019), accessed through specific entries for each organism in the NCBI Taxonomy Browser^1^, which has specific entries for each strain.

## Results

### The Relationship Between Enzymes, Proteins, and Genome Size Follows a Power-Law Distribution

**FIGURE 1:**
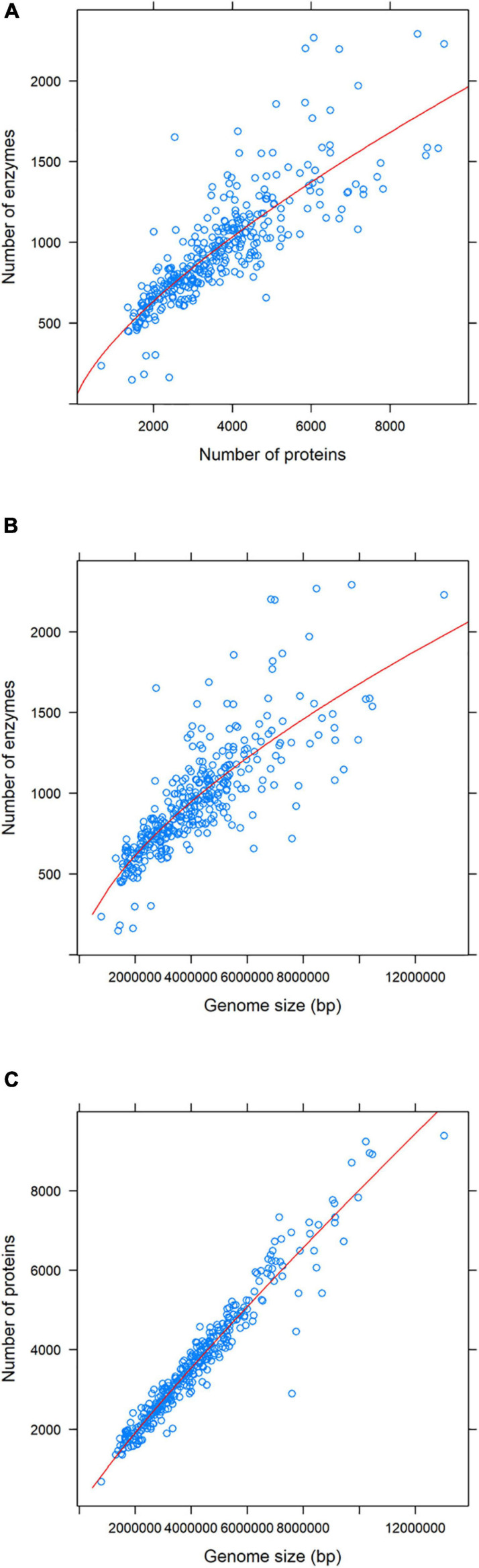
Relation between the enzyme and protein content, and the genome size. For each pair of variables, a power-law equation is the one that best explains the distribution of the data. The equations and *R*-squared values are as follows: **(A)** *y* = 2.51*x*^0.72^; *R*^2^ = 0.79, **(B)** *y* = 0.04*x*^0.66^; *R*^2^ = 0.76; **(C)** *y* = 0.004*x*^0.9^; *R*^2^ = 0.95.

Before analyzing paralogous enzymes’ content, we inspected the relationship between enzymes, proteins, and genome size. Visually, it seemed that there was a linear relationship between each pair of those variables. However, regression analyses revealed that a power-law function was the best that explained our data (Figure 1). This makes more sense for the relationship between enzymes and proteins, and for enzymes and genome size (Figures 1A,B), because there are different kinds of proteins (i.e., regulatory, structural, etc.) encoded in genes. So, as genomes grow, one does not necessarily expect that organisms accumulate a higher ratio of enzymes because that would imply that many more regulatory proteins would be needed to regulate those enzymes (Koonin and Wolf, 2008). However, one would expect a linear relationship between the number of proteins and the genome size. As Figure 1C shows, this is not precisely the case due to, perhaps, the organisms with the smallest genomes (lower-left part of the figure).

As in the previous point, we performed the same analysis with the sample divided by its lifestyle. The results are shown in Supplementary Figures 1–4. We found the same trend for variable comparison for free-living and pathogen organisms, as in Figure 1 (i.e., a power-law distribution) (Supplementary Figures 1, 3). Surprisingly, for extremophile organisms, this was not the case. In all cases, we found a linear relationship between each pair of variables (Supplementary Figure 2). It is interesting to note that this is perhaps the most homogenous group of organisms concerning genome size (most of them have a genome under six megabases (Mb), and none of them has a genome less than 1 Mb). Finally, we found a trend like that of the extremophiles for intracellular organisms, with one exception. Linear regression is what best explains the relationship between the number of enzymes and the number of proteins and genome size, although this is not the case for the relationship between proteins and genome size, which follows a power-law distribution (Supplementary Figure 4).

### The Ratio of Paralogous Enzymes Also Follows a Power-Law Distribution

**FIGURE 2:**
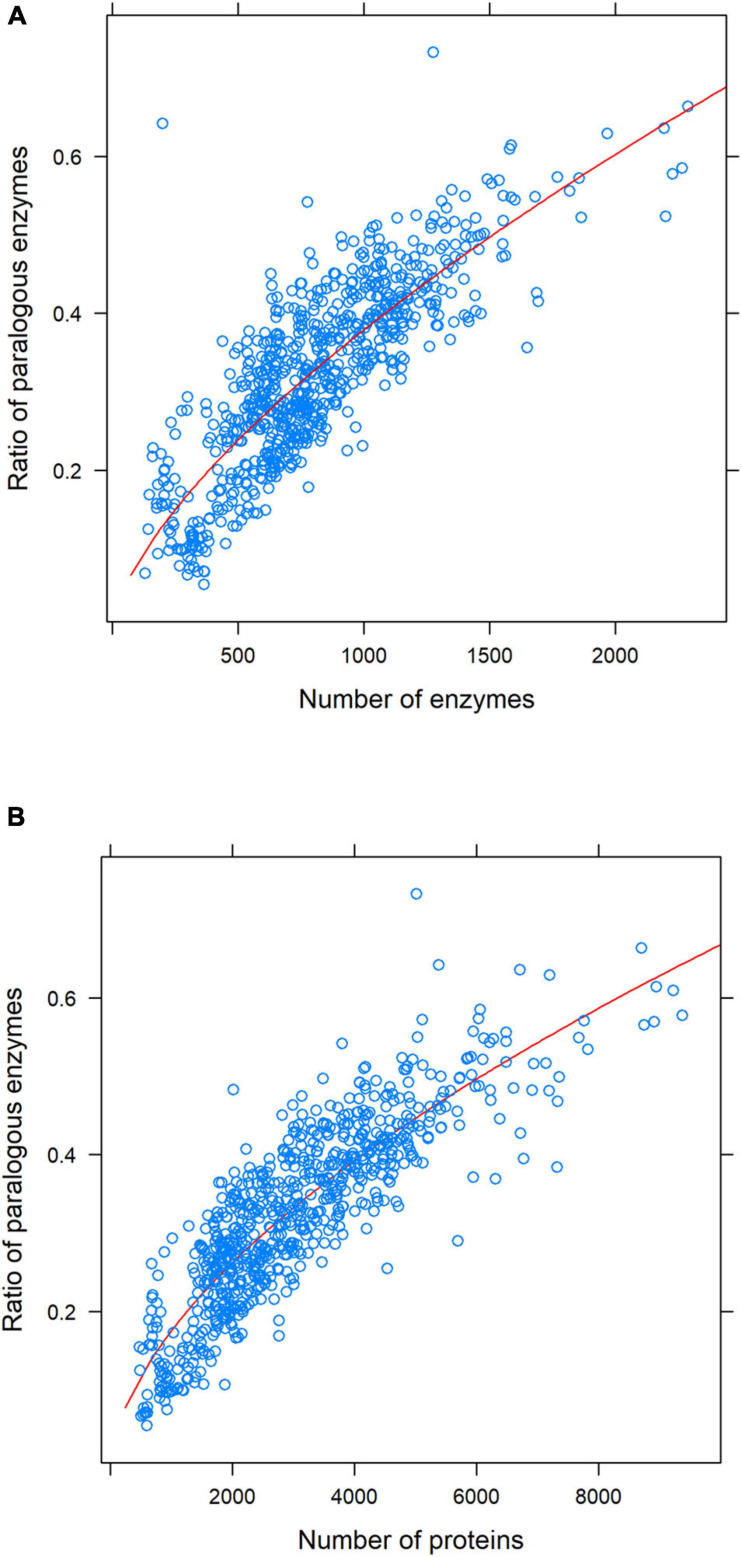
Ratio of paralogous enzymes as a function of the number of enzymes **(A)** and the number of proteins **(B)**, for each individual genome. The power-law equation for each adjustment and the *R*-squared value are as follows: **(A)** *y* = 0.003*x*^0.67^; *R*^2^ = 0.68; **(B)** *y* = 0.003*x*^0.58^; *R*^2^ = 0.75. Note that the *R*-squared value is higher when the number of proteins is considered (*R*^2^ = 0.75) instead of the number of enzymes (*R*^2^ = 0.68).

The ratio of paralogous enzymes within each proteome was calculated by dividing the number of paralogous enzymes identified in each proteome by the same proteome’s total number of enzymes. We defined as “enzymes” all those sequences that had assigned the first number of the EC code, which indicates the general function of the enzyme. We considered the ratio instead of the total number of enzymes because there was such a disparity across organisms’ whole sample. So, this was a way to eliminate the bias associated with such disparity and homogenize the data. As shown in Figure 2, the relation between those variables follows a power-law distribution (*R*^2^ = 0.68). It is noteworthy that such a ratio is less than 0.6 for most organisms (less than ten organisms have a higher ratio; their number of enzymes goes from 1000 to 2000).

### The Ratio of Paralogous Enzymes Differs Between the Different Enzymatic Classes

**FIGURE 3:**
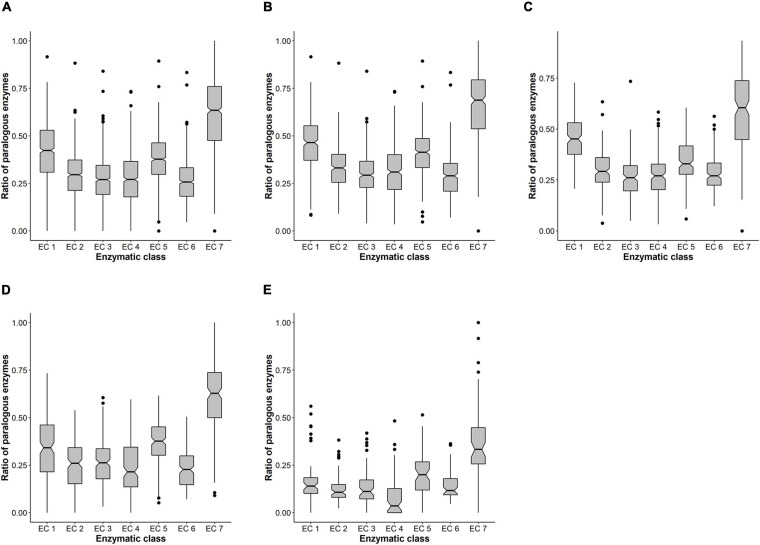
Ratio of paralogous enzymes in prokaryotic organisms. In panel **(A)**, the ratio of paralogous enzymes is plotted for the whole sample and is separated by enzymatic class. For panels **(B–E)**, we also plot the ratio of paralogous enzymes for each enzymatic class but separated by the four different lifestyles in which we sorted our initial sample: **(B)** free-living organisms; **(C)** extremophiles; **(D)** pathogenic (non-intracellular) organisms; **(E)** intracellular organisms (both endosymbiont and intracellular pathogens).

We performed a Kruskal–Wallis test to evaluate if there was any difference in the ratio of paralogs between different enzymatic classes. The *P*-value was statistically significant (*P* ≤ 2.2e-16), and so we then performed a *post hoc* Dunn test with the Bonferroni adjustment in order to identify between which classes there was a significant difference (Figure 3A and Supplementary Table 1). The α value was set at 0.05, and the *P*-value at α/2 (*P*-value = 0.025). Overall, we found three enzymatic classes whose ratio of paralogs differed significantly from all the others: the Oxidoreductases, the Isomerases, and the recently created Translocases.

We then wondered if this trend was found in different sub-samples of prokaryotic organisms or if we were detecting significant differences due to the large dataset we were considering. It has been previously reported that the number of enzymes differs significantly among different lifestyles of organisms (Martínez-Núñez et al., 2015), so we decided to investigate if the same thing also happened regarding the number of paralogs. To do so, we reclassified our sample into four sub-samples. These correspond to different lifestyles: free-living, extremophile, pathogen, and intracellular. Each organism’s lifestyle was identified using the bacterial metadatabase Bac*Dive* (Reimer et al., 2019). We performed a Kruskal–Wallis test for each of the four sub-samples, and we found significant differences in all cases. Afterward, we performed a Dunn test and obtained similar results to those of the whole sample (Figures 3B–E and Supplementary Table 2). In summary, the trend we found in the whole sample, regarding those classes with a significantly higher ratio of paralogs, is also found no matter the organisms’ lifestyle. Isolated exceptions are found in extremophiles between classes EC 1 and EC 7, for which there are no significant differences (Figure 3C); in pathogens, between EC 1 and EC 5 (they do not differ significantly) (Figure 3D); and in intracellular organisms (Figure 3E), for which the ratio of paralogous oxidoreductases and isomerases is underrepresented.

### The Ratio of Paralogous Enzymes Differs Among Lifestyles

As was noted previously, we found that some enzymatic classes have significantly higher ratios than others within each lifestyle and that this pattern, if not the same, was quite similar within each of the four lifestyles that we considered. We also wanted to know if there were any differences in the ratio of paralogs *among* the different lifestyles. A Dunn test with the Bonferroni adjustment was performed for the whole dataset to evaluate whether paralogous enzymes’ overall ratio was either the same or different when comparing the four lifestyles. The α value was set at 0.05, and *P*-value at α/2 (*P*-value = 0.025). As it is shown in Supplementary Table 3 and Supplementary Figure 5, we found significant differences among each lifestyle, and the highest ratio is found for the free-living organisms, followed by the extremophiles (both over 30%), then pathogens (less than 30 but over 20%) and, finally, intracellular organisms (less than 20%) (Supplementary Table 4).

**FIGURE 4:**
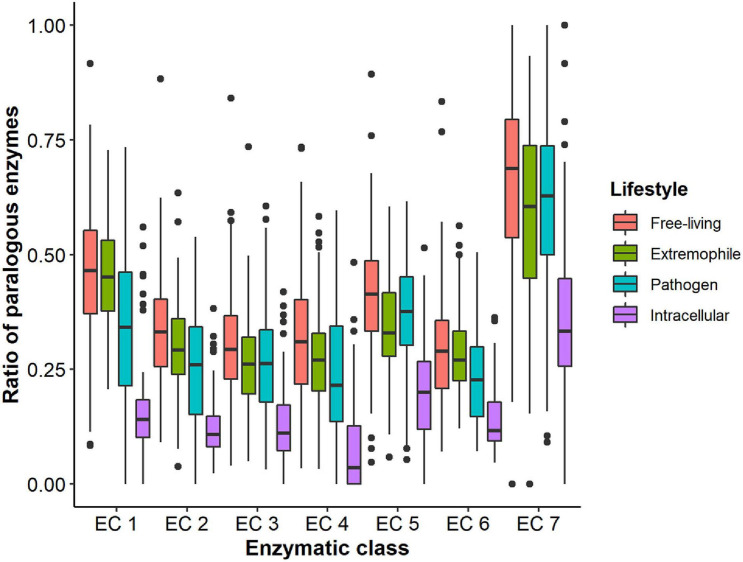
Boxplot showing the comparison of the average paralogous-enzymes ratio across the different lifestyles. Black dots represent the outliers that are found within each lifestyle. Interestingly, the lifestyle within which we found the highest number of outliers was in the intracellular organisms.

A similar approach was taken to compare each class among the four lifestyles. Although we obtained similar results to those when we analyzed the ratio without separating it by enzymatic classes, we think some exceptions are worth mentioning. These are listed below and shown in Figure 4 and Supplementary Table 5.

1. ***Oxidoreductases*.** This is one of the classes with the highest ratio values, mainly for free-living and extremophile organisms (both have a ratio higher than 40%), but there are no significant differences among them. This is the only case for this class in which ratios are not statistically significant. Their corresponding paralogs-ratio is higher than in pathogens and intracellular organisms.
2. ***Transferases.*** For this class, the ratios follow the same trend as in the whole dataset. We did not find non-significant differences.
3. ***Hydrolases*.** This class exhibits lower paralogous-enzymes ratios than the oxidoreductases and transferases. The highest ratio corresponds to free-living organisms and is roughly 30%. Extremophiles and pathogens have very similar values (26–27%), whose difference is non-significant. The intracellular have a ratio of less than 15%.
4. ***Lyases*.** For this class, the difference between the ratios was always significant. The ratio for free-living organisms is slightly higher than 30%, followed by extremophiles and pathogens (between 20 – 30%). Intracellular organisms possess the lowest ratio, which is lower than 10%. It is noteworthy that this is the lowest ratio in this group of organisms.
5. ***Isomerases*.** This is one of the enzymatic classes in which we found some of the highest ratios. For free-living organisms, the ratio is slightly higher than 40%, followed by the pathogens (37%), the extremophiles (35%), and intracellular organisms (20%). This ratio was exceptionally high for this last group and is only surpassed by that of translocases. For this enzymatic class, the only non-significant difference was found between extremophiles and pathogens.
6. ***Ligases*.** In this case, none of the ratios is higher than 30%, although in free-living, extremophile and pathogen organisms are higher than 20%. This ratio is slightly less than 15% in the intracellular organisms. For extremophile and free-living organisms, there are no significant differences.
7. ***Translocases*.** This recently created enzymatic class exhibits the highest ratios of paralogous enzymes. For all the groups but intracellular organisms, such a ratio is well over 50%, and the difference is non-significant only between pathogens and extremophiles. Even the intracellular organisms have a high ratio, slightly fewer than 40%.

Taking these results together, we can argue that the extremophiles represent perhaps the most interesting group in terms of their paralogous-enzyme content. They seem to be in-between the free-living and pathogenic organisms, sometimes very close to one or the other. This is reflected by the fact that the only five cases in which we found similar, non-significant ratios involved the extremophiles. There were non-significant differences between extremophiles and pathogens in three such cases, and the other two, between free-living organisms and extremophiles. For the intracellular organisms, the ratio difference was always the lowest (and always significantly) for each of the seven enzymatic classes.

### Detailed Exploration of the Parologous Enzymes Ratio

Our data clearly show an overrepresentation of paralogous enzymes within oxidoreductases, isomerases, and translocases. However, considering only the enzymes’ general function gives us scarce information about the patterns found within each class. This is important because there is an unequal number of subcategories within each enzymatic class, inherent to the Enzyme Commission classification system (Table 1). Furthermore, if we want to get a complete picture of the reasons underlying the high ratio of paralogs within these categories, a deeper analysis breaking down each category could be quite useful.

**TABLE 1:** Number of subcategories and entries for each enzymatic class.

Enzymatic class	EC code	No. of subclasses	No. of sub-subclasses	No. of enzymes
Oxidoreductases	EC 1	26	148	1798
Transferases	EC 2	10	38	1900
Hydrolases	EC 3	13	66	1360
Lyases	EC 4	8	17	677
Isomerases	EC 5	7	19	310
Ligases	EC 6	6	12	203
Translocases	EC 7	6	10	90

*Data as of November 2020, taken from the ExplorEnz Database (McDonald et al., 2009).*

**FIGURE 5:**
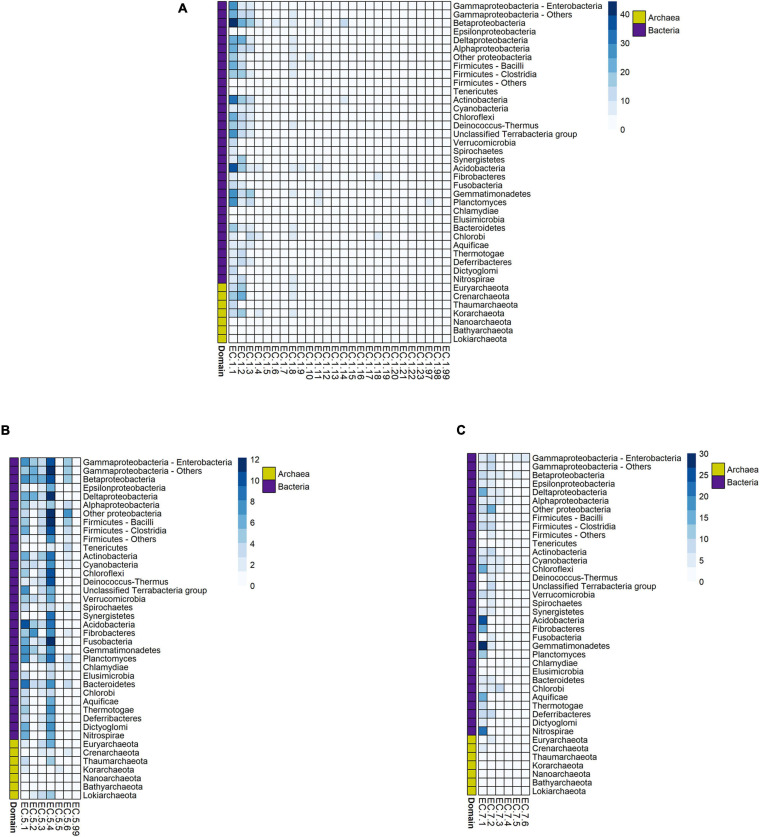
Number of paralogous enzymes found within prokaryotic oxidoreductases **(A)**, isomerases **(B)**, and translocases **(C)** subclasses. Each cell in the heatmaps represents the mean value of the phylum for that specific subclass.

We identified the number of paralogs within each of the subclasses from the above-mentioned enzymatic classes for our whole dataset. Given that this was an exploratory analysis, we considered that the average value for each individual phylum could be a good starting point. So, we averaged the number of paralogous enzymes for each subclass, and we report the values *per* phylum for each of them. The results are separated by enzymatic classes and are presented as different heatmaps (Figure 5 and Supplementary Figure 6).

Separating the data into three different heatmaps allows us to make direct comparisons within each enzymatic class. The maximum number of paralogous oxidoreductases (about 44 in Betaproteobacteria) exceeds the same value for the isomerases (about 12 in several phyla). Besides, within each enzymatic class, there is also a significant disparity in the average number of paralogs. The most extreme cases are oxidoreductases, within which subclasses EC 1.1 and EC 1.2 are the ones with the highest values, followed by EC 1.3 and EC 1.8, but to a much lesser degree. For isomerases, the subclass with the highest numbers of paralogs is EC 5.4, followed by EC 5.1. However, unlike oxidoreductases, the difference between isomerases’ subclasses is less than between oxidoreductases’ subclasses. Finally, for translocases, we found the highest ratio of paralogs for subclass EC 7.1, followed by EC 7.2. For many phyla, both subclasses exhibit similar values, though there are some cases in which EC 7.1 exceeds considerably EC 7.2.

### Phylogenetically and Lifestyle-Related Phyla Share Similar Genomic and Biochemical Traits

**FIGURE 6:**
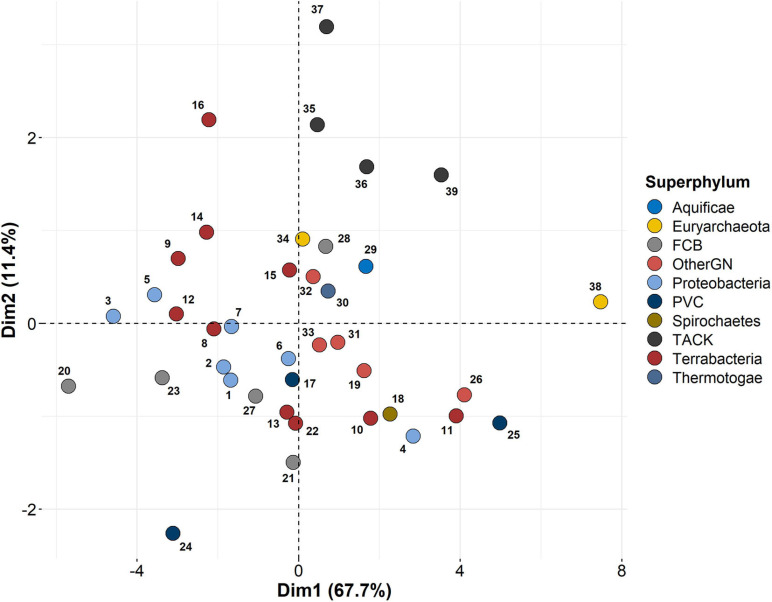
Principal component analysis, considering the mean values for the following 11 variables: genome size, number of proteins, number of enzymes, and number of paralogous proteins, plus the average ratio of paralogous enzymes for each of the seven enzymatic classes. Each circle represents a single phylum, according to the KEGG Organisms Database, and those grouped into the same superphylum are depicted with the same color. The only exceptions that aren’t included into a supergroup but considered as individual phyla are the Aquificae, Thermotogae, and Spirochaetes. Single phyla are indicated by the following numbers: (1) Gammaproteobacteria-Enterobacteria, (2) Gammaproteobacteria-Others, (3) Betaproteobacteria, (4) Epsilonproteobacteria, (5) Deltaproteobacteria, (6) Alphaproteobacteria, (7) Other proteobacteria, (8) Firmicutes-Bacilli, (9) Firmicutes-Clostridia, (10) Firmicutes-Others, (11) Tenericutes, (12) Actinobacteria, (13) Cyanobacteria, (14) Chloroflexi, (15) Deinococcus-Thermus, (16) Unclassified Terrabacteria Group, (17) Verrucomicrobia, (18) Spirochaetes, (19) Synergistetes, (20) Acidobacteria, (21) Fibrobacteres, (22) Fusobacteria, (23) Gemmatimonadetes, (24) Planctomyces, (25) Chlamydia, (26) Elusimicrobia, (27) Bacteroidetes, (28) Chlorobi, (29) Aquificae, (30) Thermotogae, (31) Deferribacteres, (32) Dictyoglomi, (33) Nitrospirae, (34) Euryarchaeota, (35) Crenarchaeota, (36) Thaumarchaeota, (37) Korarchaeota, (38) Nanoarchaeota, (39) Bathyarchaeota.

One of the main questions at the beginning of this study was whether similar organisms would share similar ratios of paralogous enzymes in terms of their phylogenetic position or lifestyle. To address this question, we performed a principal component analysis (PCA). Overall, we considered 11 variables: genome size, number of proteins, number of paralogs, number of enzymes, and the ratio of paralogous enzymes for each enzymatic class (EC 1–EC 7). As a first approach, we decided to perform this analysis with the mean values for each of these variables *per* phylum instead of individual organisms. This was due mainly to two reasons: (1) we wanted to know if there was a global pattern that might show clear differences among different phyla, and (2) given the great variation that we found for each of the eleven variables, considering individual organisms maybe would have been counterproductive, and general patterns much harder to identify. Besides, most phyla are grouped into a broader category: the superphylum. This way, it is easier to identify similarity patterns between different phyla. The only exceptions that were considered as individual phyla were the Aquificae, Thermotogae, and Spirochaetes (Supplementary Table 6) due to their lack of assignment to a superphylum. The results from the PCA are depicted in Figure 6. We decided to exclude the phylum Lokiarchaeota from the present analysis because it considerably skewed the rest of the data points (data not shown). Given that the proteome assembled for this phylum lacks a proper annotation, we think its removal from the analysis is well justified. As shown in Figure 6, the two main components explain the variation of nearly 80% of our data (PC1 = 67.7%; PC2 = 11.4%).

By taking the current approach, in which we considered the mean values *per* phylum for each of the variables, we found several interesting clusters of different phyla. The most striking result is that some phyla seem to be clustered by their lifestyle, while its phylogenetic closeness more clearly clusters others. As examples of the first type of clustering, we distinguish two main groups. One is formed by phyla whose majority of members lives in extreme or anoxygenic conditions and includes the following: Deinococcus–Thermus, Chlorobi, Aquificae, Thermotogae, Deferribacteres, Dictyoglomi, Nitrospirae, and the archaeal phylum Euryarchaeota (Figure 6, numbers 15, and 28–34). All of these belong to different superphyla. The other cluster comprises phyla in which many of its members undergo genome shrinkage due to an intracellular lifestyle. These are: Tenericutes, Elusimicrobia, and Bacteroidetes (Figure 6, numbers 11, 25, and 26). We also found two other clusters comprising closely related phyla that do not necessarily share the same lifestyle. The most remarkable case can be seen on top of the plot (Figure 6, numbers 35–37, and 39), including the Crenarchaeota, Thaumarchaeota, Korarchaeota, and Bathyarchaeota phyla. These are not only phylogenetically close, but they are all included within the TACK group of Archaea (Guy and Ettema, 2011; Spang et al., 2017). Finally, we also found that most of the proteobacteria phyla group together (Figure 6, numbers 1–3, and 5–7). The only proteobacteria phylum which is far from this group is the Epsilonproteobacteria (Figure 6, number 4) and is shown in the lower-right portion of the plot.

## Discussion

### Most Paralogous Genes in Prokaryotes Are Likely to Arise by SSD Events

The issue of LSDs and polyploidy in prokaryotes has only raised concerns until very recently. Given that in this analysis we did not make a distinction between paralogs originated by SSDs or WGDs, it could be argued that our results might be biased in some respects. Nonetheless, we do not consider this to be a severe issue.

Polyploidy does not appear to be unusual in prokaryotes (Soppa, 2011), but unlike eukaryotes, ploidy level in Bacteria and Archaea may vary depending on environmental conditions like growth rate, growth phase, among others (Breuert et al., 2006; Soppa, 2017). Besides, there does not seem to be a correlation between the ploidy level and factors such as growth temperature or lifestyle, as occurs in proteobacteria (Pecoraro et al., 2011). Having multiple genome copies could confer prokaryotes with protection against double helix breaks or serve as a phosphate reserve in phosphate-poor environments (van de Peer et al., 2017). Other benefits could be a reduction in the rate of spontaneous mutations and a way of regulating gene expression (Pecoraro et al., 2011). It has also been reported that in some of the biggest bacteria, which in many cases also have one of the largest genomes known to date, having multiple genome copies in specific parts of the cell can serve as a means of optimizing the production of locally required proteins (for example, transporters in the cell periphery) (Angert, 2012). Besides, some cultivated, monoploid bacteria could undergo one or more WGD events due to the lack of selective pressures under laboratory conditions (Soppa, 2017).

It is plausible that several organisms from our sample, either or not cultivated, have one or more copies of their entire genomes but as the evidence suggests, different genome copies are not joined together into a single chromosome but separated from each other and distributed along the cytoplasm. On the other hand, genes originated by SSDs are maintained in the bacterial chromosome until they become non-functional or acquire a function. So, when a prokaryotic genome is sequenced, it is highly likely that the obtained set of genes correspond to those located in a single genome copy and, therefore, would include only those paralogs originated by SSDs.

The presence of additional genome copies could have an impact on different kinds of studies, such as those that measure total amounts of DNA, RNA or proteins. But in our case, we think it is safe to say that we are only considering paralogous genes that are the product of SSD events, though the possibility of including in some cases ohnologs cannot be absolutely discarded.

### A Power-Law Function Explains the Relationship Between Proteins, Enzymes and Genome Size

When evaluating the relationship between proteins, enzymes, and genome size in the whole sample, we identified that the function that best fits each pair of variables was a power-law function. The most obvious cases are shown in Figures 1A,B, and involve the number of enzymes. Not all of the proteins within each genome have a catalytic function (some can be regulatory or structural proteins), and it has been shown that as prokaryotic genomes increase in size, there is an exponential growth of transcription factors (van Nimwegen, 2003) and that the opposite happens for enzymes (the larger the genome, the lower the number of enzymes/genome-size ratio) (Martínez-Núñez et al., 2013). We could say that as genomes increase their size, they also increase their protein content almost in the same proportion, which indirectly tells us that prokaryotic genomes are mainly composed of coding DNA (Koonin and Wolf, 2008). Figure 1C shows this trend, which closely resembles a linear relationship though fitting to a power-law distribution.

Regarding the ratio of paralogous enzymes, we found that it follows a power-law distribution when plotting it against the number of enzymes (*R*^2^ = 0.68) (Figure 2). For most organisms, such a ratio is between 0.2 and 0.4, which means that around 20–40% of their enzymes have at least one paralog. Congruently, most organisms with ratios lower than 0.2 are intracellular. This seems to reflect the genome reduction that happens in both endosymbionts (Wernegreen, 2015) and intracellular parasites (Sakharkar et al., 2004). It has also been shown that many intracellular organisms lose many enzymes (Price and Wilson, 2014; Manzano-Marín and Latorre, 2016). We found that the ratio of paralogous enzymes seems to reach a plateau at about 0.6. Only seven organisms exceed this value (six free-living and one extremophile), and 42 out of more than 700 organisms have a ratio higher than 0.5. One possible explanation is that there are probably more paralogs that we are not detecting with the chosen criteria. However, given that we are considering a representative sample of prokaryotes (which includes early and recently diverged lineages and some of the organisms with the largest genomes), this seems unlikely. Another more likely explanation considers the essentiality of the enzymes’ function. Although almost every gene can undergo duplication, not all of them possess the same likelihood of being retained. For example, in the eukaryote *Caenorhabditis elegans*, essential genes duplicate less often than non-essential ones but are more likely to be retained over more extended periods (Woods et al., 2013). It also has been noted that changes in the dosage of specific genes could lead to strong deleterious effects (Rice and McLysaght, 2017). However, many duplicated genes could persist if a higher gene dosage is advantageous for the organism (Kondrashov et al., 2002). Thus, one possibility is that some of the enzymes for which we found no paralogs carry out functions for which an increased dosage would result in a disruption of the metabolic flux, which in turn could compromise cell integrity. Another possibility is that, for any given query sequence, one or more of the targets are not enzymes. These are commonly known as pseudoenzymes (Jeffery, 2020). For example, Belitsky (2004) has shown that a pyridoxal 5′-phosphate(PLP)-dependent transcriptional regulator from *Bacillus subtilis* belongs to the same superfamily of a kind of PLP-dependent aminotransferases. A similar case occurs with protein kinases, which comprise one of the most diverse microbial enzyme superfamilies in terms of structure and function (Kannan et al., 2007). Phylogenetic analyses reveal that pseudokinases (that is, proteins with a kinase domain but without catalytic activity) are widely distributed throughout the tree of life (mainly in eukaryotes and bacteria) and have a pivotal, non-catalytic role in signaling processes (Kwon et al., 2019).

### High Levels of Promiscuity and Evolvability Within Oxidoreductases May Explain Their High Ratio of Paralogs

After identifying the ratio of paralogous enzymes for each enzymatic class, we noticed no clear relationship between this and the abundance of such enzymes in the genome. If this were so, one would expect that classes containing many enzymes would also show the highest ratio of paralogs. However, for the three more abundant classes (Table 1), only the oxidoreductases have a high ratio of paralogs (around 0.41), which is significantly higher than that of transferases (0.29) and hydrolases (0.27) (Figure 3). One possible explanation for this is the tremendous functional diversity within the oxidoreductases, which is reflected in the number of subdivisions within this class (Table 1). The oxidoreductases have the greatest number of subclasses amongst all enzymatic classes, and the same is observed when considering sub-subclasses. By comparing this with what is observed for the transferases, which is the class with the highest number of enzymes (Table 1), we can see that oxidoreductases’ subclasses exceed those of translocases by a factor of 2.6, whereas for sub-subclasses, it is by a factor of 3.9.

One possible explanation for why we see so much functional diversity within the oxidoreductases, which we think might also account for the high ratio of paralogs within this class, has to do with enzyme promiscuity. Promiscuous enzymatic activities are those physiologically irrelevant reactions that an enzyme can perform in addition to its native activity (Copley, 2003, 2017), and can be of two kinds: substrate promiscuity (Copley, 2020) and catalytic promiscuity (O’Brien and Herschlag, 1999). Many oxidoreductases are known to exhibit promiscuous activities of both kinds (Biegasiewicz et al., 2018; Sellés-Vidal et al., 2018); for example, the alcohol dehydrogenase of *Thermus* sp. ATN1 (TADH), which can synthesize both chiral alcohols and carboxylic acids (Höllrigl et al., 2008).

Within this enzymatic class, the highest ratios of paralogous enzymes are mainly found in subclass EC 1.1, and in subclasses EC 1.2, EC 1.3, and EC 1.8, but to a lesser degree (Figure 5A). They act upon different functional groups of their substrates; however, one common feature of these subclasses is that they contain many enzymes that utilize NAD(P)H as a cofactor. Altogether, they are the subclasses that contain the highest numbers of enzymes utilizing this cofactor, according to the CoFactor database (Fischer et al., 2010), and most of them adopt the same fold: the Rossmann fold. Phylogenetic analyses have shown that there is a common origin for proteins that share this fold, and it is likely to have been present even before the last universal common ancestor (LUCA) (Laurino et al., 2016), making it one of the most ancient protein folds (Bukhari and Caetano-Anollés, 2013; Edwards et al., 2013). Rossmann-fold proteins are also known to show high levels of evolvability, i.e., the ability to adopt new functions and to accommodate sequence changes along evolutionary time (Tóth-Petróczy and Tawfik, 2014). This capacity, along with their high levels of promiscuity (Sellés-Vidal et al., 2018), may provide an advantage for the organism (Khersonsky and Tawfik, 2010) but could also compromise the native activity of the enzyme, leading to detrimental effects. Thus, gene duplication and further optimization of the secondary function through selection could improve the new activity leading to two paralogous enzymes (Force et al., 1999).

### Unique Paralogous-Gene Retention Patterns Within the Isomerases

For the isomerases, we identified two subclasses with a high ratio of paralogs: EC 5.1 and EC 5.4 (Figure 5B). The intramolecular transferases’ subclass (EC 5.4) is also the one with the highest number of unique entries among all isomerases’ subclasses. Within it, there also exist clusters of enzymes with similar chemistries, as represented by oxidosqualene cyclases, RNA-pseudouridine synthases, and carbon mutases (Martínez Cuesta et al., 2016). Oxidosqualene cyclases comprise the biggest group of isomerases catalyzing the same kind of reaction, but although there is substantial evidence of gene duplication within this group of enzymes (Xue et al., 2012; Dahlin et al., 2016; Busta et al., 2020), the paralogous isomerases that we found are unlikely to belong to it. This is because oxidosqualene cyclases are involved in sterols and triterpenes biosynthesis, a typical eukaryotic pathway. It has been identified in several bacterial groups (Wei et al., 2016), but it is more widely considered to be a trait associated with the transition from prokaryotes to eukaryotes (Chen et al., 2007). Thus, it is more likely that paralogous enzymes belonging to this subclass are associated with different biochemical roles. It is also possible that their paralogs perform functions other than isomerization, considering that isomerases are a unique class in which changes of the primary function along their evolutionary history are widespread (Martínez Cuesta et al., 2014, 2015).

Evidence of the previous point is found within the racemases and epimerases (EC 5.1), which is the other subclass for which we found an overall high number of paralogous sequences (Figure 5B). It contains different members belonging to the subfamily of short-chain dehydrogenases/reductases (SDR), which also includes oxidoreductases (EC 1) and lyases (EC 4); all their members act upon nucleoside diphosphate (NDP) sugars (Martínez Cuesta et al., 2014). Furthermore, as it occurs with the oxidoreductases’ subclasses with more paralogs, all members of the SDR subfamily share the Rossmann fold (Jörnvall et al., 1995). It thus seems likely that, as it happens with oxidoreductases, the high evolvability of enzymes with this fold (Tóth-Petróczy and Tawfik, 2014) may explain the high number of paralogs. Additional support for this comes from several bacterial strains in which there have been identified different gene-duplication events within the SDR subfamily (Serres et al., 2009).

### Paralogous Translocases Reflect Adaptation to Different Environmental Conditions

Overall, translocases make up a unique enzymatic class because all its members come from other enzymatic classes. There are 90 different entries identified in the ExplorEnz database (McDonald et al., 2009) as of November 2020, and it is noteworthy that more than half of these entries (around 50) used to be included in a single hydrolases’ sub-subclass: EC 3.6.3, which contains enzymes acting on acid anhydrides to catalyze the transmembrane movement of substances. Most of these enzymes are ABC transporters, which constitute one of the most ancient protein superfamilies, are represented throughout both prokaryotes and eukaryotes (Saurin et al., 1999), and most likely were present in the Last Common Ancestor (Davidson et al., 2008). Within the ABC superfamily, there have been many duplication events (Saier and Paulsen, 1999; Higgins, 2001), which may be one of the reasons why we observe a high ratio of paralogous translocases (0.62), which indeed is the highest of all classes (Figure 3).

Throughout all prokaryotic diversity, ABC transporters are equally essential and classified into two main groups: uptake and efflux systems. The former plays a very important role in the nutrition of organisms because they allow direct acquisition of nutrients (Ren and Paulsen, 2005; Nicolás et al., 2007). On the other hand, efflux ABC transporters are involved in the exporting of molecules that are toxic to the organism (Nicolás et al., 2007; El-Awady et al., 2017). In the present study, we found that free-living organisms possess the highest ratio of paralogous translocases (0.67), followed by pathogens (0.62), extremophiles (0.59), and finally, intracellular organisms (0.38) (Figure 4). The only case in which we didn’t find significant differences was between pathogens and extremophiles. For both lifestyles, ABC transporters play a crucial role, though due to different reasons. Extremophiles usually live in environments where nutrients are scarce, so having a high ratio of paralogous transporters must be a good strategy for the uptake of both organic molecules and ions (Albers et al., 2001). Pathogens, rely on different kinds of transporters (including the ABC-type) to ensure the uptake of nutrients necessary for pathogenesis (Tanaka et al., 2018), and in some cases, different types of ABC transporters are active at different stages of it (Murphy et al., 2016). Again, for this group of organisms, having many paralogous translocases seems to be an adaptation for the kind of environment in which they live.

However, for intracellular organisms, we also expected a high ratio of paralogs for this class of enzymes, given the fact that they depend mainly on the uptake of nutrients from the host. Although it is the highest ratio compared to the other enzyme classes within the group, this is not the case compared to the ratios found in other lifestyles. One reason that may account for this could have to do with the kind of intracellular organisms that we considered. When comparing different groups of these organisms, Ren and Paulsen (2005) found that those associated with plants and soil environments have many more transporters than other intracellular organisms. However, in our present study, only four plant symbionts were considered, which could explain why we found a relatively low ratio of paralogous transporters compared to the other lifestyles. Nonetheless, such a ratio is still significantly higher than that of the other categories (Figure 4), which indirectly shows the importance of this class of enzymes for the intracellular lifestyle (Rodriguez and Smith, 2006).

In terms of subclasses, we found the highest ratio of paralogous translocases within subclass EC 7.1 (Figure 5C), which contains enzymes that catalyze the movement of protons across membranes. Of these, only a few contain the ATP-binding domain, so it seems unlikely that most of the paralogs found within this subclass belong to the ABC transporters. Nonetheless, many of these paralogous proteins could be involved in ATP biosynthesis. One remarkable example is the ATP synthase (EC 7.1.2.2), which is widely distributed across prokaryotes. It has been postulated that a series of several gene duplication events may have occurred earlier in the evolution of this family (Cross and Taiz, 1990), and in fact, more than one copy of ATP synthase has been found in different prokaryotic organisms (Klenk et al., 1997; Ruppert et al., 2001). Thus, many of these copies could have retained their original function, which may be related to an additional dosage requirement and would provide a benefit in terms of gene expression, given the importance of this enzyme. That this is a common trend across many distinct prokaryotic groups could be interpreted as a means of adaptation to different environments (Cross and Müller, 2004).

### Phylogenetic Proximity and Lifestyle Are Reflected in the Content of Paralogous Enzymes

Despite performing a PCA with the mean values for each phylum instead of considering each organism separately, we found different clusters of phylogenetically and lifestyle-related phyla. This was very interesting, given the high heterogeneity that exists within many different phyla. The most significant cluster comprises phyla associated with extreme environments and includes five bacterial and one archaeal phylum (Figure 6). Among these, we found two of the bacterial phyla known to have diverged earlier in bacterial evolution: Aquificae and Thermotogae. The other ones are considered lately diverging groups. This clustering suggests that there might be some genomic and biochemical constraints for organisms that inhabit hyperthermophilic environments. This notion of common features concerning lifestyles is also shown in a smaller cluster, comprising Tenericutes, Chlamydia, and Elusimicrobia phyla. All of them include many obligate intracellular organisms, which are known to have reduced genomes and incomplete metabolic pathways, as mentioned above. Although it is not known if intracellular organisms of different phyla share losses of the same (or very functionally similar) enzymes, most of them usually retain proteins involved in the uptake and internalization of organic nutrients (Saier and Paulsen, 1999) and some inorganic ions (Wandersman and Delepelaire, 2004).

Besides the clustering of phyla that share a similar lifestyle, we also found two cases of phylogenetically close phyla that cluster together. The first one comprises members of the so-called TACK group, which includes different phyla belonging to the Archaea domain. The Crenarchaeota, Thaumarchaeota, Korarchaeota, and Bathyarchaeota belong to this archaeal group (Guy and Ettema, 2011), though it includes additional phyla for which there are no fully sequenced genomes. Although belonging to the same phylogenetic group, each of these four phyla lives in different environmental conditions (Spang et al., 2017). We also found that almost all proteobacterial phyla cluster near each other in the PCA plot (Figure 6; numbers 1–3 and 5–7). As it is shown, this cluster also seems to include non-proteobacterial phyla, which we think might be due to the great physiological diversity found within the Proteobacteria as a single group (Woese, 1987), as well as the sharing of environmental conditions with other phyla like Actinobacteria and Verrucomicrobia, particularly regarding soil bacteria (Janssen et al., 2002). The only proteobacteria phylum that is far from this cluster is the Epsilonproteobacteria (Figure 6; no. 4). Recently, it has been proposed that this phylum might not be related to the other proteobacteria but constitutes an independent, monophyletic group (Waite et al., 2017). This might be reflected in genomic and biochemical traits, as our analysis suggests.

## Conclusion

In this study, we analyzed the ratio of paralogous enzymes according to the EC classification system established by the IUBMB almost 60 years ago, and that had remained without major changes until the second half of the year 2018. Around this time, a new enzymatic class was added, the translocases, consisting of enzymes previously assigned to other classes. Taking this as a starting point, we found that the number of paralogs within each enzymatic class does not always depend on the number of enzymes. Oxidoreductases are the second class with the most entries and contain many paralogous enzymes, most of which are likely to be NAD(P)H dehydrogenases that adopt the Rossmann fold. On the other hand, isomerases and translocases have, on average, the lowest number of entries but show a high ratio of paralogous enzymes. For translocases, we identified that many paralogous enzymes could be involved in ATP biosynthesis or belong to the ABC transporter superfamily. These influx/efflux systems are critical in several environmental conditions, and their diversification could be a way of adapting to new environments.

Isomerases represent a unique case for which it has been quite difficult to explain their high paralogs’ ratio. One possibility is that several paralogous sequences are not even isomerases at all but belong to other enzymatic classes (such as chemically different enzymes that are part of the SDR subfamily), as has been identified elsewhere (Martínez Cuesta et al., 2014, 2015). Additional analyses beyond the subclass level could shed more light on why isomerases have a high ratio of paralogs.

The lifestyle of organisms also seems to be related to the content of paralogous enzymes. Free-living organisms have the highest ratio of paralogs for all enzymatic classes, whereas extremophiles and pathogens have similar ratios, and for certain classes, they do not differ significantly. On the other hand, intracellular organisms show the lowest ratios. However, this trend could be due to other variables like genome size or the number of proteins. Further statistical analysis could help to identify the most important factors determining the prevalence of a high ratio of paralogous enzymes in different organisms.

By considering the ratios of paralogous enzymes and other aspects of the genome, we found a clustering of several phyla not only in a phylogenetic but also in a similar-lifestyle context. The most striking example was a group of different phyla whose members share a hyperthermophilic lifestyle. Thus, it seems that a high ratio of certain paralogous enzymes could be useful to cope with this extreme environment. Whether it is due to the same enzymes, or different enzymes belonging to the same class, it is something that our current analysis did not reveal. However, evidence suggests that parts of the biochemical repertoire, like several amino acid biosynthetic pathways, could have evolved independently in different lineages (Hernández-Montes et al., 2008).

To our concern, this study is the first to analyze the content and ratio of paralogous enzymes both in terms of the EC number (considering its recent major update) and taking into account the lifestyle of organisms. Our results support the idea that gene duplication in prokaryotes is a fundamental process to cope with new environmental conditions (Gevers et al., 2004; Bratlie et al., 2010; Copley, 2020), regardless of organisms’ lifestyles.

## Data Availability Statement

The original contributions presented in the study are included in the article/Supplementary Material, further inquiries can be directed to the corresponding author.

## Author Contributions

Both authors listed have made a substantial, direct and intellectual contribution to the work, and approved it for publication.

## Conflict of Interest

The authors declare that the research was conducted in the absence of any commercial or financial relationships that could be construed as a potential conflict of interest.

## Footnotes

1 https://www.ncbi.nlm.nih.gov

## Supplementary Material

Supplementary Figure 1 Relation between the enzyme and protein content, and the genome size in free-living organisms. For each pair of variables, a power-law equation is the one that best explains the distribution of the data. The equations and *R*-squared values are as follows: **(A)** *y* = 3.02*x*^0.7^; *R*^2^ = 0.7; **(B)** *y* = 0.07*x*^0.63^; *R*^2^ = 0.65; **(C)** *y* = 0.005*x*^0.89^; *R*^2^ = 0.94.

Supplementary Figure 2 Relation between the enzyme and protein content, and the genome size in extremophile organisms. For each pair of variables, a linear regression equation is the one that best explains the distribution of the data. The equations and *R*-squared values are as follows: **(A)** *y* = 0.18*x* + 247; *R*^2^ = 0.72, **(B)** *y* = 1.46e^–04^*x* + 306; *R*^2^ = 0.78; **(C)** *y* = 7.65e^–04^*x* + 457; *R*^2^ = 0.96.

Supplementary Figure 3 Relation between the enzyme and protein content, and the genome size in pathogen organisms. For each pair of variables, a power-law equation is the one that best explains the distribution of the data. The equations and *R*-squared values are as follows: **(A)** *y* = 2.39*x*^0.73^; *R*^2^ = 0.83; **(B)** *y* = 0.02*x*^0.71^; *R*^2^ = 0.82; **(C)** *y* = 0.002*x*^0.95^; *R*^2^ = 0.97.

Supplementary Figure 4 Relation between the enzyme and protein content, and the genome size in intracellular organisms. For panels **(A,B)** (number of enzymes vs. number of proteins, and number of enzymes vs. genome size), a linear equation is the one that best explains the distribution of the data. This is not the case for panel **(C)**, in which the data fits best to a power-law equation. The equations and *R*-squared values are as follows: **(A)** *y* = 0.25*x* + 95; *R*^2^ = 0.84; **(B)** *y* = 1.54e^–04^*x* + 180; *R*^2^ = 0.71; **(C)** *y* = 0.02*x*^0.79^; *R*^2^ = 0.88.

Supplementary Figure 5 Comparison of the ratio of paralogous enzymes across the different lifestyles. **(A)** The ratio for each organism is plotted together with its number of proteins and enzymes. Each of the four colors represents organisms from the same lifestyle. The diameter of each point of the plot is proportional to the ratio of paralogous enzymes, as indicated in the right part of the figure. **(B)** Notched box plots for the average ratio of paralogous enzymes for the organisms grouped by its lifestyle. Graphically, the ratio value differs significantly in all cases because the notches never overlap each other.

Supplementary Figure 6 Number of paralogous enzymes found within prokaryotic oxidoreductases **(A)**, isomerases **(B)**, and translocases **(C)** subclasses. Each cell of the heatmaps represents the mean value of the phylum for that specific subclass. The values were scaled for each column using the formula *z* = (*x* − *u*)/*s*, where *x* is the unscaled value, *u* is the mean of each column, and *s* is the column’s standard deviation.
