# Supplementary material for: The Role of Gene Duplication in the Divergence of Enzyme Function: A Comparative Approach
Source: Front Genet. 2021 Jul 14;12:641817. doi: 10.3389/fgene.2021.641817 (PMC8318041; doi:10.3389/fgene.2021.641817)
Supplement: Supplementary Figure 1 — Relation between the enzyme and protein content, and the genome size in free-living organisms. For each pair of variables, a power-law equation is the one that best explains the distribution of the data. The equations and R-squared values are as follows: (A) y = 3.02x0.7; R2 = 0.7; (B) y = 0.07x0.63; R2 = 0.65; (C) y = 0.005x0.89; R2 = 0.94. [file Data_Sheet_1.DOCX]

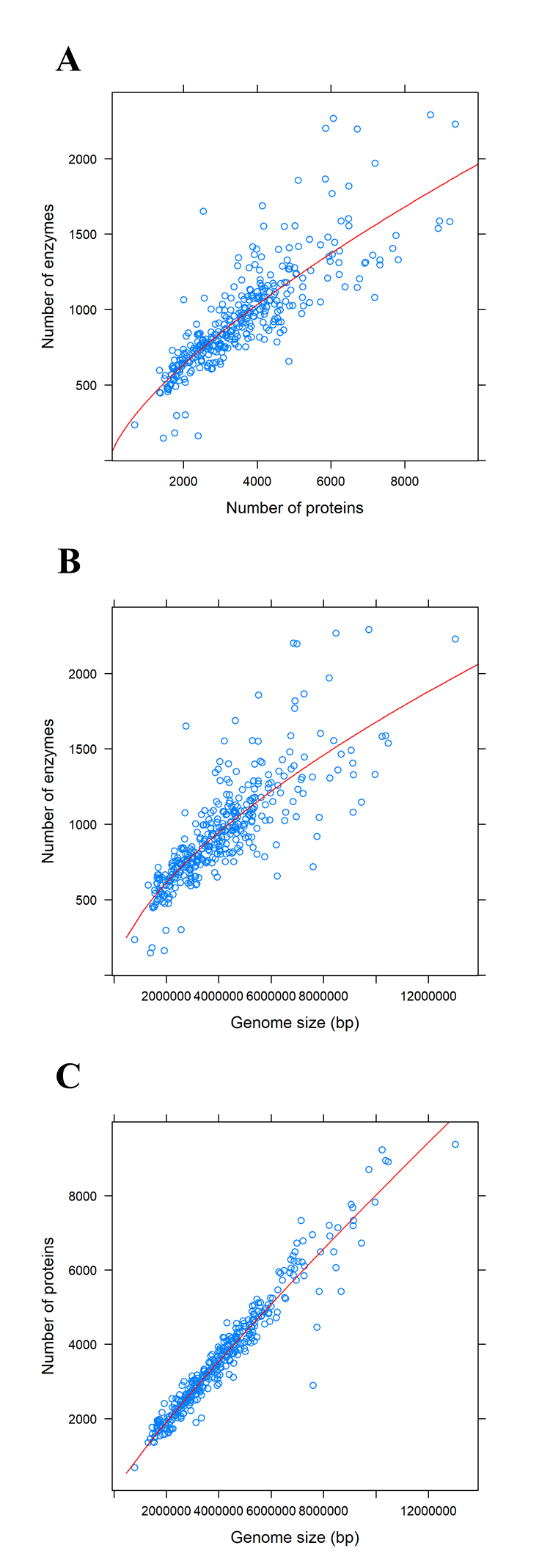


**Figure S1.** Relation between the enzyme and protein content, and the genome size in free-living organisms. For each pair of variables, a power-law equation is the one that best explains the distribution of the data. The equations and *R*-squared values are as follows: A) *y*=3.02*x*^0.7^; *R*^2^=0.7; B) *y*=0.07*x*^0.63^; *R*^2^=0.65; C) *y*=0.005*x*^0.89^; *R*^2^=0.94.


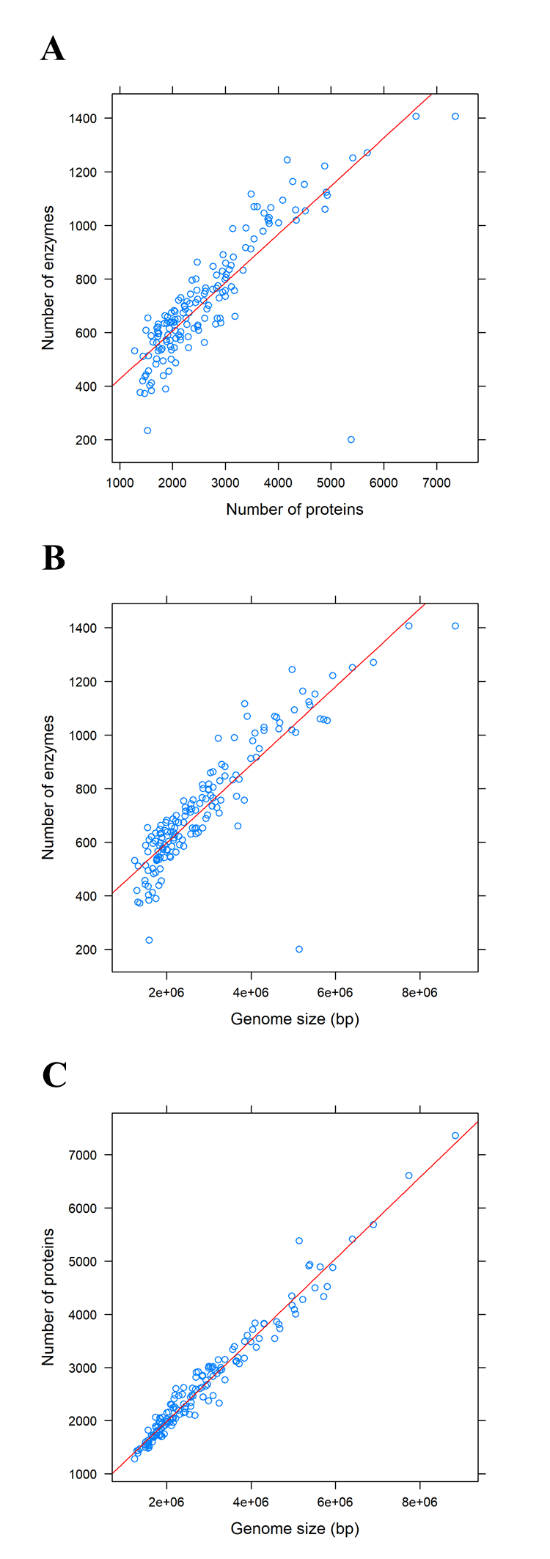


**Figure S2.** Relation between the enzyme and protein content, and the genome size in extremophile organisms. For each pair of variables, a linear regression equation is the one that best explains the distribution of the data. The equations and *R*-squared values are as follows: A) *y*=0.18*x* + 247; *R*^2^=0.72, B) *y*=1.46e^-04^*x* + 306; *R*^2^=0.78; C) *y*=7.65e^-04^*x* + 457; *R*^2^=0.96.


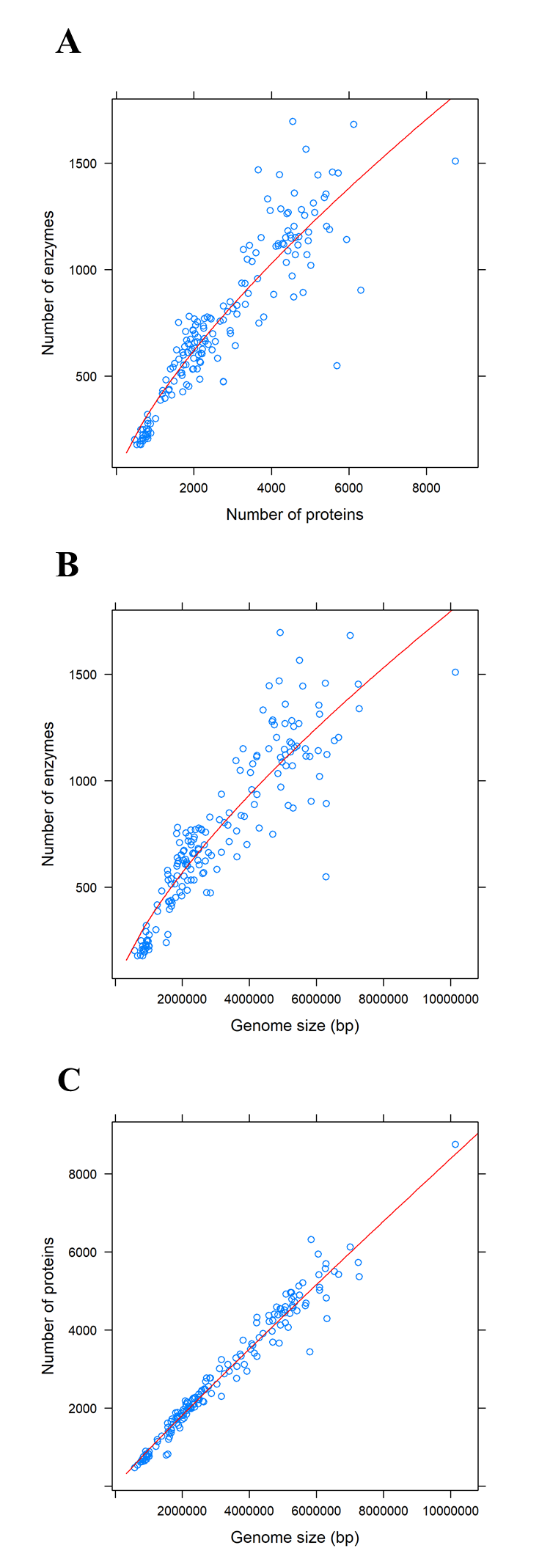


**Figura S3.** Relation between the enzyme and protein content, and the genome size in pathogen organisms. For each pair of variables, a power-law equation is the one that best explains the distribution of the data. The equations and *R*-squared values are as follows: A) *y*=2.39*x*^0.73^; *R*^2^=0.83; B) *y*=0.02*x*^0.71^; *R*^2^=0.82; C) *y*=0.002*x*^0.95^; *R*^2^=0.97.


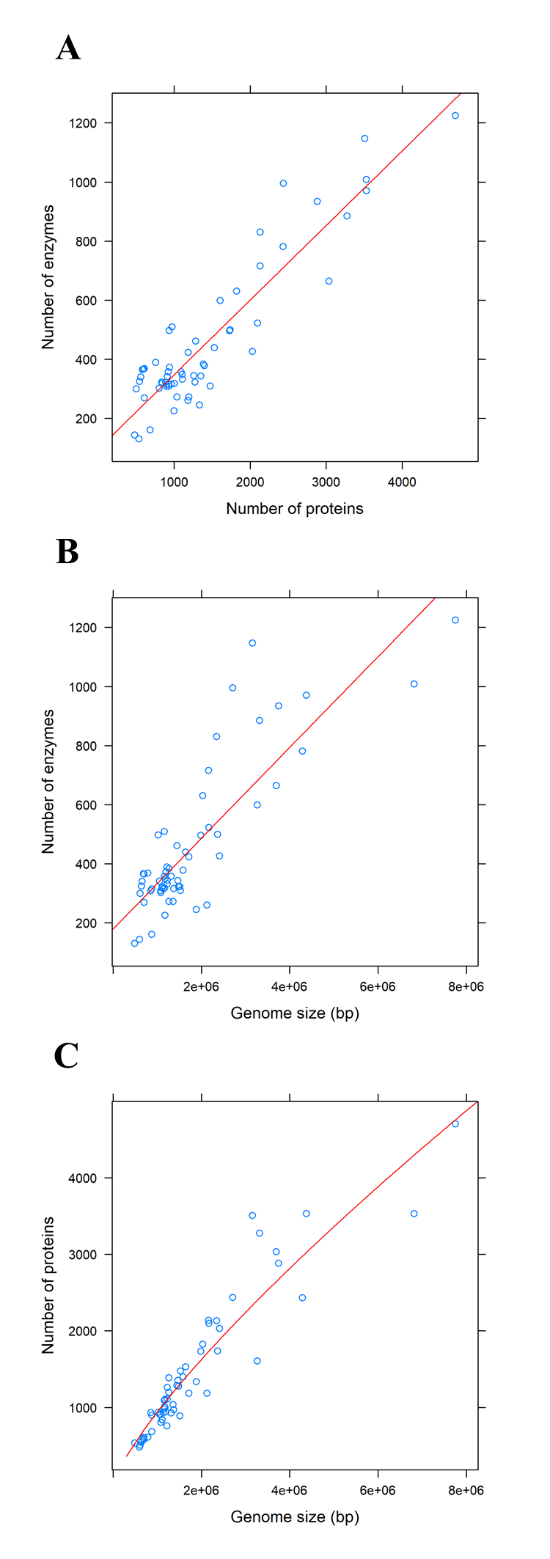


**Figure S4.** Relation between the enzyme and protein content, and the genome size in intracellular organisms. For A and B (number of enzymes vs number of proteins, and number of enzymes vs genome size), a linear equation is the one that best explains the distribution of the data. This is not the case for C, in which the data fits best to a power-law equation. The equations and *R*-squared values are as follows: A) *y*=0.25*x* + 95; *R*^2^=0.84; B) *y*=1.54e^-04^*x* + 180; *R*^2^=0.71; C) *y*=0.02*x*^0.79^; *R*^2^=0.88.


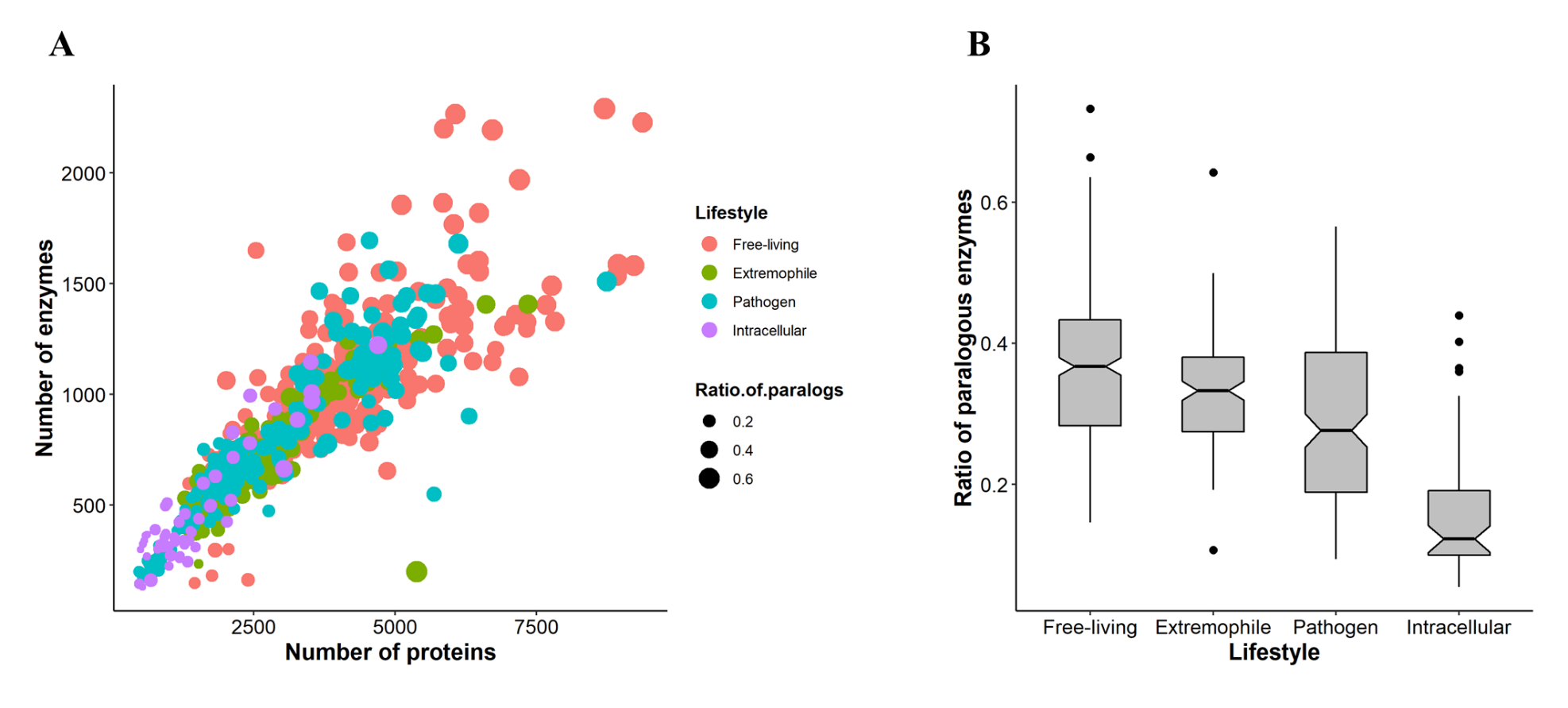


**Figure S5.** Comparison of the ratio of paralogous enzymes across the different lifestyles. (A) The ratio for each organism is plotted together with its number of proteins and enzymes. Each of the four colors represents organisms from the same lifestyle. The diameter of each point of the plot is proportional to the ratio of paralogous enzymes, as indicated in the right part of the figure. (B) Notched box plots for the average ratio of paralogous enzymes for the organisms grouped by its lifestyle. Graphically, the ratio value differs significantly in all cases because the notches never overlap each other.


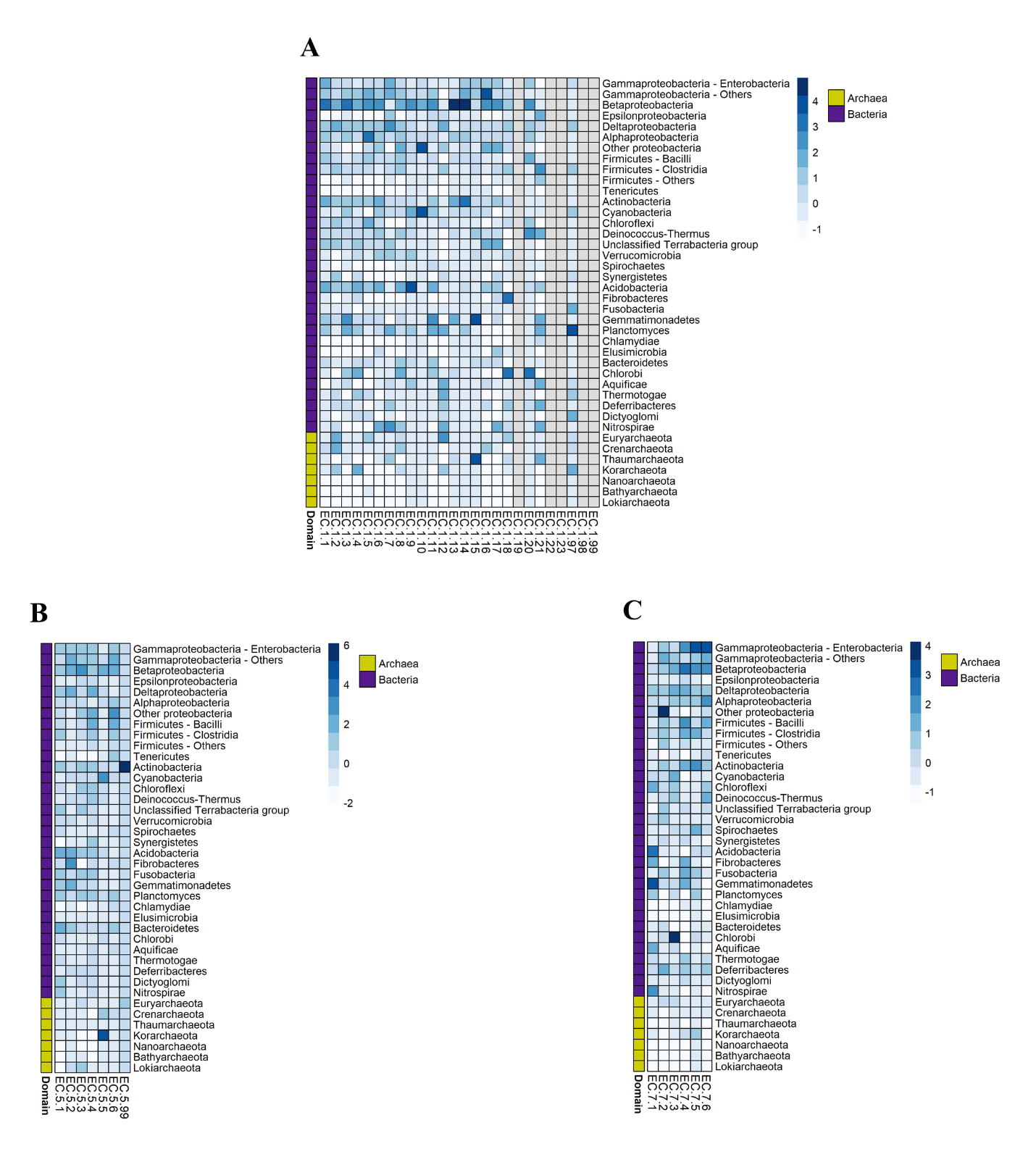


**Figure S6.** Number of paralogous enzymes found within prokaryotic oxidoreductases (A), isomerases (B), and translocases (C) subclasses. Each cell of the heatmaps represents the mean value of the phylum for that specific subclass. The values were scaled for each column using the formula z = (x - u) / s, where *x* is the unscaled value, *u* is the mean of each column, and *s* is the column’s standard deviation.
