# Supplementary material for: The Role of Gene Duplication in the Divergence of Enzyme Function: A Comparative Approach
Source: Front Genet. 2021 Jul 14;12:641817. doi: 10.3389/fgene.2021.641817 (PMC8318041; doi:10.3389/fgene.2021.641817)
Supplement: Supplementary file 3 [file Data_Sheet_3.docx]

# Supplementary tables

**Table S1.** *P*-values from the Dunn test for the comparisons of paralogs’s ratio among the seven enzymatic classes. We took into account the whole sample. The *P*-value is defined as α/2, which is equivalent to 0.025. Non-significant values are indicated with double asterisk (**).

|  | EC 3 | EC 5 | EC 6 | EC 4 | EC 1 | EC 2 |
| --- | --- | --- | --- | --- | --- | --- |
| EC 5 | 0.0000** |  |  |  |  |  |
| EC 6 | 1.0000 | 0.0000** |  |  |  |  |
| EC 4 | 1.0000 | 0.0000** | 1.0000 |  |  |  |
| EC 1 | 0.0000** | 0.0085** | 0.0000** | 0.0000** |  |  |
| EC 2 | 0.0517 | 0.0000** | 0.0006** | 0.1554 | 0.0000** |  |
| EC 7 | 0.0000** | 0.0000** | 0.0000** | 0.0000** | 0.0000** | 0.0000** |

**Table S2.** *P*-values from the Dunn test for the comparisons of paralogs’s ratio among the seven enzymatic classes. In this case, organisms with similar lifestyles were sorted in one of four subgroups. The *P*-value is defined as α/2, which is equivalent to 0.025. Non-significant values are indicated with double asterisk (**).

|  |  | EC 3 | EC 5 | EC 6 | EC 4 | EC 1 | EC 2 |
| --- | --- | --- | --- | --- | --- | --- | --- |
| **Free-living** | EC 5 | 0.0000** |  |  |  |  |  |
|  | EC 6 | 1.0000 | 0.0000** |  |  |  |  |
|  | EC 4 | 1.0000 | 0.0000** | 0.2080 |  |  |  |
|  | EC 1 | 0.0000** | 0.0014** | 0.0000** | 0.0000** |  |  |
|  | EC 2 | 0.0228** | 0.0000** | 0.0007** | 1.0000 | 0.0000** |  |
|  | EC 7 | 0.0000** | 0.0000** | 0.0000** | 0.0000** | 0.0000** | 0.0000** |
| **Extremophile** | EC 5 | 0.0000** |  |  |  |  |  |
|  | EC 6 | 1.0000 | 0.0000** |  |  |  |  |
|  | EC 4 | 1.0000 | 0.0000** | 1.0000 |  |  |  |
|  | EC 1 | 0.0000** | 0.0000** | 0.0000** | 0.0000** |  |  |
|  | EC 2 | 0.0632 | 0.0171** | 1.0000 | 1.0000 | 0.0000** |  |
|  | EC 7 | 0.0000** | 0.0000** | 0.0000** | 0.0000** | 0.0965 | 0.0000** |
| **Pathogen** | EC 5 | 0.0000** |  |  |  |  |  |
|  | EC 6 | 0.2094 | 0.0000** |  |  |  |  |
|  | EC 4 | 1.0000 | 0.0000** | 1.0000 |  |  |  |
|  | EC 1 | 0.0011** | 0.0726 | 0.0000** | 0.0000** |  |  |
|  | EC 2 | 1.0000 | 0.0000** | 1.0000 | 1.0000 | 0.0001** |  |
|  | EC 7 | 0.0000** | 0.0000** | 0.0000** | 0.0000** | 0.0000** | 0.0000** |
| **Intracellular** | EC 5 | 0.0601 |  |  |  |  |  |
|  | EC 6 | 1.0000 | 0.2261 |  |  |  |  |
|  | EC 4 | 0.0102** | 0.0000** | 0.0018** |  |  |  |
|  | EC 1 | 1.0000 | 1.0000 | 1.0000 | 0.0001** |  |  |
|  | EC 2 | 1.0000 | 0.0174** | 1.0000 | 0.0371 | 1.0000 |  |
|  | EC 7 | 0.0000** | 0.0000** | 0.0000** | 0.0000** | 0.0000** | 0.0000** |

**Table S3.** *P*-values from the Dunn test for the comparisons of paralogs’s ratio among different lifestyles. In this case, a significant *P*-value is defined as α/2, which is equivalent to 0.025.

|  |  | **Lifestyle** | | |
| --- | --- | --- | --- | --- |
|  |  | Extremophile | Free-living | Intracellular |
|  | Free-living | 0.0131 |  |  |
| **Lifestyle** | Intracellular | 0.0000 | 0.0000 |  |
|  | Pathogen | 0.0031 | 0.0000 | 0.0000 |

**Table S4.** Average ratio of paralogous enzymes according to the different lifestyles. The standard deviation and standard error for each group are also indicated.

| **Lifestyle** | **Average ratio** | **Standard deviation** | **Standard error** |
| --- | --- | --- | --- |
| Free-living | 0.365 | 0.101 | 0.005 |
| Extremophile | 0.333 | 0.078 | 0.006 |
| Pathogen | 0.289 | 0.114 | 0.009 |
| Intracellular | 0.155 | 0.088 | 0.011 |

**Table S5.** *P*-values from the Dunn test for the comparisons of paralogs’s ratio among different lifestyles. In this case, the *P*-value is defined as α/2, which is equivalent to 0.025. Non-significant values are shown in italics.

| **Enzymatic class** | **Lifestyle** | | | |
| --- | --- | --- | --- | --- |
|  |  | Extremophile | Free-living | Intracellular |
|  | Free-living | *1.0000* |  |  |
| Oxidoreductases | Intracellular | 0.0000 | 0.0000 |  |
|  | Pathogen | 0.0000 | 0.0000 | 0.0000 |
|  | Free-living | 0.0052 |  |  |
| Transferases | Intracellular | 0.0000 | 0.0000 |  |
|  | Pathogen | 0.0074 | 0.0000 | 0.0000 |
|  | Free-living | 0.0005 |  |  |
| Hydrolases | Intracellular | 0.0000 | 0.0000 |  |
|  | Pathogen | *1.0000* | 0.0007 | 0.0000 |
|  | Free-living | 0.0164 |  |  |
| Lyases | Intracellular | 0.0000 | 0.0000 |  |
|  | Pathogen | 0.0193 | 0.0000 | 0.0000 |
|  | Free-living | 0.0000 |  |  |
| Isomerases | Intracellular | 0.0000 | 0.0000 |  |
|  | Pathogen | *0.0738* | 0.0036 | 0.0000 |
|  | Free-living | *1.0000* |  |  |
| Ligases | Intracellular | 0.0000 | 0.0000 |  |
|  | Pathogen | 0.0000 | 0.0000 | 0.0000 |
|  | Free-living | 0.0002 |  |  |
| Translocases | Intracellular | 0.0000 | 0.0000 |  |
|  | Pathogen | *0.6449* | 0.0000 | 0.0000 |

**Table S6.** The prokaryotic supergroups. The Aquificae, Thermotogae and Spirochaetes are not associated with a specific supergroup, the reason why we considered these phyla individually.

| **Superphylum** | **Phyla included within each superphylum** |
| --- | --- |
| Proteobacteria | Gammaproteobacteria – Enterobacteria, Gammaproteobacteria – Others, Betaproteobacteria, Epsilonproteobacteria, Deltaproteobacteria, Alphaproteobacteria, Other proteobacteria |
| Terrabacteria | Firmicutes – Bacilli, Firmicutes – Clostridia, Firmicutes – Others, Tenericutes, Actinobacteria, Cyanobacteria, Fusobacteria, Chloroflexi, Unclassified Terrabacteria group, Deinococcus - Thermus |
| FCB | Gemmatimonadetes, Fibrobacteres, Bacteroidetes, Chlorobi, Acidobacteria |
| PVC | Chlamydiae, Verrucomicrobia, Planctomyces |
| Other Gram-negative bacteria | Elusimicrobia, Deferribacteres, Dictyoglomi, Synergistetes, Nitrospirae |
| - | Aquificae |
| - | Thermotogae |
| - | Spirochaetes |
| Euryarchaeota | Euryarchaeota, Nanoarchaeota |
| TACK | Crenarchaeota, Korarchaeota, Thaumarchaeota, Bathyarchaeota |
